# King Edward's Hospital Fund for London

**Published:** 1918-12-28

**Authors:** 


### The Hospital

The Workers' Newspaper of Administrative Medicine and Institutional Life, Administration, National Insurance and Health.

No. 1699, Vol. LXV. SATURDAY, DECEMBER 28, 1913. PRICE TWOPENCE

### KING EDWARD'S HOSPITAL FUND FOR LONDON.

This Fund has attained its majority, and the meeting of its Governors and general council at St. James's Palace on the 17th instant had consequently special interest. It was largely attended and the discussions displayed keen interest and thoroughness. Lord Revelstoke, the honorary treasurer, in announcing that the amount to be distributed at that meeting was £200,000, stated that the total receipts on general fund to December 7, after deducting expenses, amounted to £174,531, and the estimated total receipts for the whole year were £178,271. To this had to be added the yield from the League of Mercy £16,000, being an increase of £1,000 on the previous year, making £194,271. Lord Revelstoke was able to report that the Fund had with its bankers or at short call sufficient cash to meet the whole £200,000 distributed. This is highly satisfactory, remembering that the period dealt with is the last of the war years, when the claims upon all classes of the public for monetary gifts have been abnormal and excessive. Further, it must be borne in mind that His late Majesty King Edward VII., in his original letter, when Prince of Wales, inaugurating the Fund on the 5th February, 1897, mentioned the goal which the Fund must aim at was a distribution of £100,000 to £150,000 a year.

The success of this Fund has been remarkable. The investments standing in the name of King Edward's Hospital Fund for London on the 31st December, 1917, and taken at values adjusted as at the 21st July 1908, in accordance with the terms of King Edward's Hospital Fund for London Act, 1907, and in the case of securities subsequently received or acquired at or under the market price at the date of gift or purchase, were £2,454,677. The Fund also had in cash at banks and short call £57,748. We are confident that every one interested in the continuance and welfare of the London hospitals, and who is not? will wish the Fund on its attaining its majority, and all connected with it, long life and continued prosperity. It has saved the London hospitals and rendered it possible to add immeasurably to their efficiency during the twenty-one years of its existence and useful work.

It is of the first importance that the League of Mercy, which was founded by King Edward VII. in order to raise in small sums of 10s. and under, £50,000 a year in free gifts from the healthy as an act of thanksgiving to Almighty God for the blessings of health, should continue to depend for its revenue on this principle which has made the League unique amongst agencies established to raise funds for the hospitals. This principle must be maintained at the forefront of its programme, if the League is to continue to pursue its useful purpose, and to maintain its progress and extend its yield in continuous years. In a statement made on behalf of the League at the King's Fund meeting on the 17th inst. by Sir William Collins it was shown that in twenty years of its existence, including the £16,000 for the King's Fund in 1918, the League had contributed £276,000 to King Edward's Fund, and to the extra-metropolitan hospitals in the area of its collection, outside the area of distribution of the King's Fund, £29,280, making for the twenty years a total contribution from the League of Mercy to the voluntary hospitals, in and around London, of £305,280. This is an encouraging and most satisfactory result of the League's work. Sir William Collins further stated that the total contribution from the League during the five years of war was £74,000, that is an average of £14,800 a year as against £14,000 in the last pre-war year, and an average of £13,500 in all the pre-war years. The last figure, £13,500, is misleading, for it includes the first five years during which the League's organisation had to be created, the first year being for a broken period when the King's Fund received but £1,000. The annual sum from the League increased year by year until the fifth year, 1903, when the grant to the King's Fund was £10,000, and the average for the first five years was £6,400. The League then settled down to work, and the average contribution to King Edward's Fund during the next ten pre-war years, including 1913, was not £13,500 but £17,000 yearly.

In three out of the last five years, including the last two, the League of Mercy receipts were swollen in 1915 by entertainments which yielded £1,908, in 1917 £2,476, and in 1918, according to the treasurer's statement (see page 259), by profits made at a concert, by a legacy and by the sum from a trust, amounting in all to some £2,100. We hope that the Prince of Wales's assumption of the office of Grand President will create a new and keen interest in the principle of the League, and that the main yield of the League in future will come through personal service as free gifts, in gratitude for the blessings of health. The whole hospital world will rejoice to have H.R.H. the Prince of Wales as President of the King's Hospital Fund, in which he is bound to take the keenest interest, and to which H.R.H. will no doubt bring fresh energy and inspiration. The advent of the new President cannot fail to result in added benefit and increased interest on the part of the people for the voluntary hospital system everywhere throughout the country. A debt of gratitude is due from the hospitals to the three Governors of the Fund for eminent services during the minority of H.R.H.

